# Cohort Profile: the Cambridge Baby Growth Study (CBGS)

**DOI:** 10.1093/ije/dyv318

**Published:** 2015-12-31

**Authors:** Philippa Prentice, Carlo L. Acerini, Antigoni Eleftheriou, Ieuan A. Hughes, Kenneth K. Ong, David B Dunger

**Affiliations:** ^1^ Department of Paediatrics, University of Cambridge, Cambridge, UK and; ^2^ Medical Research Council Epidemiology Unit, University of Cambridge, Cambridge, UK

## Why was the cohort set up?

The Cambridge Baby Growth Study (CBGS) is a prospective, observational pregnancy and birth cohort with detailed assessments and sample collections in infancy and continuing childhood follow-up. Women were recruited during early pregnancy from April 2001 to March 2009; they attended several study visits throughout pregnancy, and then repeatedly with their infants until the age of 2 years. The main aim of the study has been to investigate the antenatal and postnatal determinants of infancy growth and reproductive development, including environmental, genetic, hormonal and nutritional exposures. Collection of extensive anthropometric, nutritional, demographic and biological data has allowed detailed examination of the first 1000 days of life, a time of emerging importance for the early origins of health and disease, where *in utero* exposures, early growth, nutrition and genetic interactions are thought to contribute to later-life disease risk, ^1–3^

All women were recruited from a single study site: the Rosie Maternity Hospital, Cambridge, UK, and the Cambridge local research ethics committee approved the study. Initial funding was from the European Union Framework V, with the specific objective to investigate the effect of environmental factors during pregnancy on male offspring reproductive development over the first 2 years of life. From the outset, the study was designed to also include female offspring and to collect data on infancy growth and body composition. Subsequent funding and collaborations have allowed further nested case control studies and continuing follow-up through childhood. Cohort funding has been received from the World Cancer Research Fund International, the Newlife Foundation for Disabled Children, the Mothercare Group Foundation and a collaborative research grant from the European Society for Paediatric Endocrinology. Support from the Medical Research Council (MRC) has been awarded through a clinical research training fellowship (G1001995), a Cambridge University MRC Centenary award, and assisted by MRC Department of Epidemiology programmatic funding (U106179472). Lipidomic assays were also supported by the MRC (UD99999906) and the Cambridge Lipidomics Biomarker Research Initiative (G0800783). Genotyping and placental RNA work was funded by grants from the Evelyn Trust, the Wellbeing of Women and Diabetes UK (11/0004241). Funding from Mead Johnson Nutrition has allowed further biochemical analysis of biological specimens, in particular breast milk samples, and childhood follow-up. Continued support and funding are provided by the National Institute of Human Research Cambridge Comprehensive Biomedical Research Centre.

## Who is in the cohort?

Women were recruited during early pregnancy, at approximately 12 weeks of gestation, at their first routine antenatal ultrasound clinic in the Rosie Maternity Hospital, Cambridge, UK. At the time, the hospital had approximately 5000 deliveries per year from a mixed urban and rural community mainly residing in South Cambridgeshire. Research nurses introduced details of the study and distributed information sheets. Those who showed interest in the study were followed up by telephone and the first study visit was arranged, where informed written consent was provided. Exclusion criteria included women under the age of 16 years, or those unable to give informed consent.

**Figure 1.**
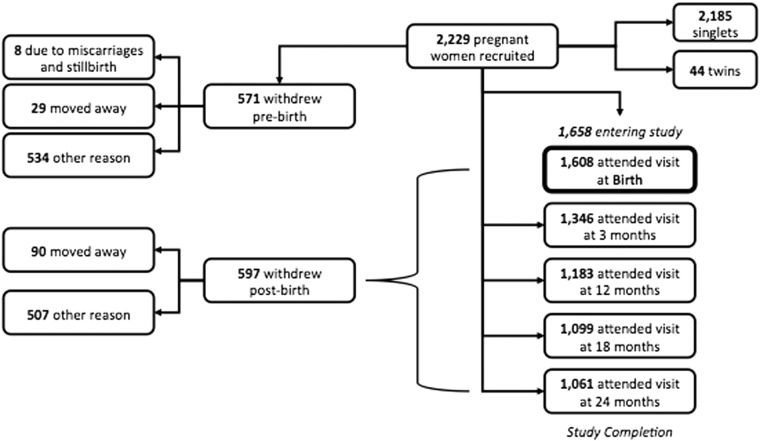
Participant numbers from recruitment and the infancy study protocol. Singlets, singletons.

Figure 1 shows the numbers of participants accrued and followed up throughout the study period: 2229 women were recruited in early pregnancy (∼ 50% of those who were approached for participation) and 1658 offspring were studied (including 22 sets of twins), following additional written consent from the mother on behalf of her infant(s). Of these infants, 1183 were seen at 1 year of life and 1061 completed the infancy study protocol at age 2 years.

Offspring who were followed until at least 1 year of age, and are now aged between 5 and 10 years, are currently being invited for a follow-up study visit. At the time of writing, 423 children have been invited out of a total of 917 eligible children, with a 44% participation rate.

Table 1 describes the characteristics of the CBGS cohort. Demographic data were not available for non-participating women. However, the recruited term and late preterm (gestational age > = 36 weeks) mother-infant dyads were similar to the overall population of births at the Rosie Maternity Hospital, as shown in Table 2 , albeit CBGS mothers were slightly older and more likely to be primiparous.

**Table 1.** Cohort characteristics ( *N* indicates the total sample with available data)

Continuous variables	*N*	Mean (SD)	Categorical variables	*N*	% (with outcome specified)
Parental			Ethnicity	1619	
Maternal age (years)	1338	33.5 (4.3)	White Caucasian		95.3
Paternal age (years)	1291	35.7 (5.4)	Black		1.3
Maternal height (cm)	1237	165.9 (7.2)	Asian		1.7
Maternal pre-pregnancy weight (kg)	1203	66.3 (12.4)	Other		1.7
Maternal pre-pregnancy BMI (kg/m ^2^ )	1173	24.1 (4.6)			
Maternal pregnancy weight gain (kg)	866	8.2 (6.7)	Maternal education	780	
Index of multiple deprivation	920	8.9 (4.2)	University degree-level		62.7
			Other		37.3
			Smoking during pregnancy	1575	5.5
			Maternal weight	1173	
			Overweight (BMI > 25)		30.6
			Obese (BMI > 30)		9.9
			Primiparous pregnancy	1629	43.4
			Recorded diabetic pregnancy	1562	7
Birth					
Gestational age	1638	39.8 (1.6)			
Birthweight (kg)	1657	3.47 (0.55)	Small for gestational age	1657	1.1
Newborn length (cm)	1599	51.4 (2.6)	Large for gestational age	1657	2.7
Newborn head circumference (cm)	1602	35.3 (1.7)			
Newborn mean skinfold thickness (mm)	1599	6.3 (1.6)	Delivery (caesarian delivery)	1630	30.2
			Infant sex (male)	1660	51.7
Infancy					
3-month weight (kg)	1340	6.15 (0.83)	Rapid infancy weight gain 0–12 months	1179	
3-month length (cm)	1335	61.1 (2.6)	Catch-up weight gain		26
3-month head circumference (cm)	1342	40.7 (1.4)	Normal weight gain		46.7
3-month mean skinfold thickness (mm)	1342	10.8 (2.0)	Catch-down weight gain		27.3
12-month weight (kg)	1182	9.97 (1.19)			
12-month length (cm)	1175	75.9 (2.9)			
12-month head circumference (cm)	1179	46.4 (1.4)			
12-month mean skinfold thickness (mm)	1178	11.3 (2.1)			
18-month weight (kg)	1094	11.36 (1.32)			
18-month length (cm)	1096	82.2 (3.2)			
18-month head circumference (cm)	1088	47.8 (1.5)			
18-month mean skinfold thickness (mm)	1096	10.8 (2.1)			
24-month weight (kg)	1051	12.61 (1.47)			
24-month length (cm)	1043	87.8 (3.4)			
24-month head circumference (cm)	1051	48.7 (1.5)			
24-month mean skinfold thickness (mm)	1057	10.3 (2.2)			
Infancy nutrition					
Age stopped breastfeeding (months)	436	5.3 (3.1)	3-month feeding	1319	
Age started solids (months)	652	5.0 (0.9)	Exclusively breastfed		42.2
			Mixed-fed		24.9
			Exclusively formula-fed		29
			S'tarted solids		3.9

**Table 2.** The Cambridge Baby Growth Study cohort compared with all infants born at term (gestational age ≥ 36 weeks) at the Rosie Maternity Hospital in 2005 (midway through the study).Mean (SD) or % are shown

	CBGS	Rosie Maternity Hospital
	*N* = 1585	*N* = 4670
Birthweight (kg)	3.52 (0.49)	3.47 (0.49)
Gestational age (weeks)	40.0 (1.3)	40.0 (1.3)
Maternal age	33.5 (4.3)	30.7 (5.6)
Caucasian ethnicity	95%	91%
Spontaneous vaginal delivery	60%	60%
Primiparous	43%	22%
Index of multiple deprivation	9.0	11.5 (Cambridgeshire)

## How often have they been followed?

The study protocol included visits across antenatal, perinatal and postnatal periods, with a wide range of data collection ( Table 3 ).

**Table 3.** Study schedule

	Pregnancy (weeks)					Infancy (months)				Childhood (years)		Father
	12	28	36	Birth	6 weeks	3	12	18	24	3–4	5–10	
**Physical measures**												
Anthropometry				•		•	•	•	•		•	
Anogenital distance				•		•	•	•	•			
Abdominal ultrasound						•	•		•		•	
DXA scan											•	
Pubertal assessment											•	
Blood pressure	•	•	•								•	
**Biological Samples**												
Venous blood	•											
OGTT		•									•	
Cord blood and placenta				•								
Dried blood spot						•	•	•	•			
Breast milk					•							
DNA sample	•			•								•
**Questionnaires**												
Perinatal			•									
Postnatal						•						
Temperament						•						
3-day food diary							•		•			
Pre-school activities inventory										•		

### 

#### Pregnancy Visits

Antenatally, women were seen during each trimester of pregnancy. This included an early pregnancy maternal venous blood sample at approximately12 weeks of gestation for biochemical analytes and DNA extraction, a 75-g oral glucose tolerance test (OGTT) at 28 weeks and administration of a questionnaire on parental demographics and environmental exposures, including diet and chemical exposure, which was returned in late pregnancy.

#### Birth visit

Cord blood and placental samples were collected at birth where possible. Birthweight was recorded at delivery by midwives and obtained from the hospital notes. Infants were also examined as newborns, as early as possible in the first 2 weeks of life, either in hospital or at home visits, with further anthropometric measurements as detailed below. Infant DNA was extracted from the cord blood sample, where available.

#### Infancy visits

Infants were seen at research clinic visits at 3, 12, 18 and 24 months of age. These visits included detailed longitudinal anthropometric measurements and questionnaires on feeding, behaviour and illness. Biological samples consisted of repeated dried blood spot collection, breast milk samples and infant venous or mouth swab samples for DNA when cord blood was not available. A mouth swab was also obtained from the father. Clinics were held in two locations due to the large geographical catchment area of the Rosie Maternity Hospital: at the Cambridge Biomedical Campus (the NIHR-Wellcome Trust Clinical Research Facility or the MRC Epidemiology Clinical Research Facility) and at the Princess of Wales Hospital, Ely.

Mothers and infants who completed the initial follow-up to 2 years of age were very similar to those lost to follow-up, although they were slightly older and more likely to be of Caucasian ethnicity ( Table 4 ).

**Table 4.** Mother-infant dyads who completed infancy assessments to age 2 years ( *N* = 1061) compared with those infants lost to follow-up ( *N* = 597)

	Followed-up	Lost to follow-up
	Mean (SD)	Mean (SD)
Birthweight (kg)	3.50 (0.54)	3.44 (0.55)
Gestational age (weeks)	39.8 (1.6)	39.7 (1.7)
Maternal age (years)	33.7 (4.3)	32.8 (4.6)
Caucasian ethnicity	97%	92%
Spontaneous vaginal delivery	58%	60%
Primiparous	43%	43%
Index of multiple deprivation	8.9 (3.6)	9.0 (2.2)
Exclusively breastfed at 3 months	43%	40%

#### Childhood visit

A Pre-school Activities Inventory (PSAI) questionnaire was posted to a subgroup of 194 mothers of 3–4-year-old children in 2010–11 (with 80% response rate). Children aged 5–10 years are currently being re-recruited into a follow-up phase.

Throughout the study period, families have been kept informed of study updates and activities, with biannual posted or e-mailed newsletters, and through two study events for participating families in March 2010 and May 2011.

## What has been measured?

### Anthropometry

A team of four trained paediatric research nurses carried out all the measurements. Infancy anthropometry included weight and length, and skinfold thickness measurements at four sites (triceps, subscapular, flank, quadriceps). In September 2006, abdominal ultrasound assessment of visceral and subcutaneous-abdominal fat depths was introduced into the protocol ( ***N*** = 498 at 3 months; ***N*** = 582 at 12 months, ***N*** = 550 at 24 months), as well as waist circumference measurement. This novel ultrasound method was validated in infants against magnetic resonance imaging (MRI). ^4^ All anthropometric measures are currently being repeated at the childhood follow-up visit, with the addition of dual-energy X-ray absorptiometry (DXA) scans.

Anogenital distance (AGD) was measured using a modified version of a previously published method, ^5^ defined as the distance from the centre of the anus to:the junction of smooth perineal skin and rugated skin of the scrotum in boys; and the lower vaginal opening in girls, as well as penile length in boys, using Vernier calipers.Measurement of AGD was added to the CBGS protocol in 2006.

For all physical measurements, there were regular training and quality control sessions and the technical error of measurements (TEM) within and between observors was calculated. Over the study period **,** the relative intraobserver TEM ranged **as follows** : for infant length **,** between 0.03 and 0.50%; for individual skinfold thickness measurements, between 0.4 **and** 2.8%; for intra-abdominal depth, between 0.3 **and** 1.7%; and for subcutaneous abdominal fat depth, between 1.1 **and** 2.6%. Relative interobserver TEM were: for infant length, 0.7%; for individual skinfold thickness measurements, 2.0 **to** 3.2%; for intra-abdominal depth, 3.2%; and for subscutaneous abdominal fat depth, 3.6%. Interobserver TEM for AGD was 9.6% for males and 5.7% for females.

### Biological specimens

During pregnancy, a non-fasting venous blood sample was collected from mothers at approximately 12 weeks **of** gestation, into endocrine disrupting chemicals (EDC) contamination-free glass containers. EDCs, including phthalate metabolites and phenols of mothers with singleton male pregnancies have been measured using liquid chromatography tandem mass spectrometry. A fasting 75 g-OGTT was completed at 28 weeks **of** gestation in 1071 women. Cord blood and placental tissue were collected when possible. RNA was extracted from placental tissues ( *N* = 420). Genomic DNA was extracted from cord blood samples, maternal and/or infancy blood samples or mouth swabs, and paternal mouth swabs for single nucleotide polymorphism(SNP) genotyping. A total of 845 trios have been extracted and used in analyses.

Dried blood spot samples (DBS) were collected repeatedly between 3 to 24 months of age. Capillary heel-prick blood sampling was used, and single drops of blood were added to untreated filter paper (Ahlstrom 226, ID Biological Systems), and stored at −20°C. For analysis, 3.2-mm single spots were punched from the larger DBS. Since DBS have not been widely used, work was done to investigate effects of storage conditions and time, ^6^^,^^7^ and to validate assays converted from plasma assays—for example IGF-1, other hormones, metabolites and lipidomic profiles. ^7^^,^^8^

Mothers who breastfed their infants were asked to hand-express a pooled hindmilk samplebetween 4 and 8 weeks postnatally. After feeding their infant, theyexpressed from the same breast as they had last fed their infant from, and repeated this process several times, so that a total of 100 mls of hindmilk was collected over a **2-** week period, in order to reduce within-day and day-to-day variations. **A total of** 641 pooled breast milk samples were collected and stored at −20°C, until processed at a single time point.

### Questionnaires

Several questionnaires were administered. The perinatal questionnaire, completed in late pregnancy, included questions on socio-demographic status, self-reported maternal (pre-pregnancy) and paternal anthropometry, medications,environmental exposures **and** dietary and medical information. At 3 months of infancy age, the postnatal questionnaire included questions on infant feeding practice (exclusive breastfeeding, mixed feeding or exclusive formula feeding), health and illnesses. The revised infant behaviour questionnaire was used to assess three main dimensions of infant temperament. ^9^

At the 12-month and 24-month clinic visits, a 3-day diet diary was collected. Dietary data, measured using household measures (teaspoon, cup etc.), age-appropriate portion sizes and home-made food recipes, were then coded by trained dietary assessment assistants using the Human Nutrition Research (HNR) in-house programme DINO (Diet in Nutrients Out), based on McCance and Widdowson *The Composition of Foods,* 6th **e** dition. ^10^ The DINO output generated a food code and weight for each food item recorded. From this information, total energy intake was calculated, as well as dietary intake of fat, saturated fat, protein, carbohydrate and specific micronutrients.

## What has been found? Key findings and publications

Findings from CBGS to date have mainly focused on the antenatal and early postnatal determinants of infancy body size, male genital development and exposures influencing maternal metabolism. We have shown that DBS are a useful biological medium and many serum assays can be adapted, allowing measurement of early -ife circulating hormones, metabolites and lipids. Our findings have therefore expanded current knowledge around infancy growth and physiology, related to both prenatal and early postnatal exposures, in particular those of nutrition. Work with DBS assays has begun to fill a gap in the literature with respect to infancy hormones and metabolites, from studies on cord blood to those in childhood. Additionally, further knowledge of maternal and infancy metabolism may allow biomarkers for fetal and infancy growth. Our main findings from the cohort are summarized below.

### Antenatal determinants of infancy size and maternal metabolism

It is clear that antenatal determinants of infant size include environmental *in utero* exposures and genetic influences, but also their interactions. We have shown that maternal fasting glucose levels and maternal pre-pregnancy BMI are both independently positively related to offspring adiposity in the non-diabetic population. ^11^ Whereasaternal glycaemia was correlated with infant adiposity at birth, but not afterwards, maternal pre-pregnancy BMI showed increasingly strong associations with offspring adiposity as infants aged.

#### Fetal imprinted genes

We demonstrated that fetal SNP genotypes at the paternally-imprinted (maternally-expressed) gene *H19* were associated with offspring size at birth. ^12^ Fetal genotypes at paternally-expressed imprinted genes were also found to influence birthweight, likely indirectly by affecting maternal metabolism. Fetal genotypes at the paternally-expressed gene *IGF2* (encoding insulin-like growth factor-2) were associated with maternal glucose levels during pregnancy, ^13^ and preliminary work in the CBGS has also suggested that polymorphic variation in various paternally-expressed fetal imprinted genes may also affect maternal blood pressure. ^14^

### Infancy nutrition

#### Metabolic markers of infancy nutrition

Three-month insulin-like growth factor 1 (IGF-1) levels were analysed in 566 DBS samples and found to be higher in formula-fed infants than in breastfed infants. ^8^ Extensive work using high-resolution mass spectrometry has also demonstrated striking differences between the DBS lipidomic profiles of breastfed and formula-fed infants at 3 months of age ( *N* = 241); differences were evident across all main lipid classes, with mixed-fed infants showing intermediate profiles. ^15^ Breastfed infants had higher levels of cholesterol esters, lower total phosphatidylcholines (PC) and specific differences in many individual PCs and sphingomyelin lipid species.

#### Age of weaning

Age of weaning (introduction of semi-solid or solid foods) was inversely related to infancy weight and length, but these associations are likely to be due to reverse causality. ^16^ Consistent with evidence synthesized across other studies, these findings suggest that infants who grow faster are more likely to be weaned earlier than other infants, but that in turn age at weaning has neutral effects on infant growth.

#### Feeding practice and infant behaviour

Maternally-rated infancy temperament differed between breast/mixed-fed and formula-fed infants at 3 months of age. ^17^ Breastfed and mixed-fed infants were rated as having more challenging temperaments, with lower soothability, greater distress and less positive response to stimulation. These results may allow better understanding and expectation of normal infant temperament and this knowledge, with appropriate support, could be used to promote successful breastfeeding.

### Regulation of postnatal growth

#### Hormones

We demonstrated that infant DBS IGF-1 levels at age 3 months predicted subsequent faster linear growth and lesser gains in adiposity, up to the age of 12–18 months. ^8^ It is likely that other hormones mediate the faster gains in adiposity seen in contemporary milk formula-fed infants, and we are currently investigating several hormones, including leptin, which are positively associated with weight and adiposity in infancy. Preliminary work has also suggested that several specific lipid species in the infancy lipidome are related to 3- and 12-month weight. ^15^

#### Genes

An individual-level meta-analysis of CBGS and three other European birth cohorts showed that adult obesity-risk alleles were not associated with birth size, but were positively associated with body size at 1 year, with strengthening correlations seen across the first 5 years of life. ^18^ These alleles were not only associated with fat mass, as seen in older populations, but had a symmetrical influence on both body size and body composition (lean and fat mass). Of note, these alleles showed less influence on infants born with low or high birthweights than on those of average birthweight.

#### Epigenetics

DNA methylation patterns have been measured in selected sub-groups—infants of mothers with gestational diabetes and infants demonstrating rapid postnatal weight gain over the first year of life—and compared with a group of control infants. We found multiple differentially methylated sites, including at or near genes with known relevance to growth and diabetes, and several methylation differences that were common to infants of mothers with gestational diabetes and infants who showed rapid infancy weight gain. ^19^ This work suggests that similar epigenetic changes may occur after different *in utero* exposures, and may possibly contribute to the same long-term phenotype, for example with increased risk of obesity and type 2 diabetes.

### Male reproductive development

CBGS is one of the largest cohort studies with repeated measurements of infancy penile length, testicular descent and anogenital distance (AGD) (a marker of androgen exposure). The study has generated normative data for infancy anogenital distance, for both boys and girls. ^20^ AGD increased from birth up to age 12 months in a sexually-dimorphic pattern, and was positively correlated with penile length at birth. These normative data will be useful as markers of androgen action in further studies investigating environmental effects on genital development. CBGS data also showed a higher incidence of congenital cryptorchidism than reported in earlier UK studies. ^21^ We are currently investigating a possible role for EDCs in male reproductive disorders, exploring data on maternal first-trimester serum phthalate and phenol levels. We are also exploring how EDCs affect maternal metabolism during pregnancy.

## What are the main strengths and weaknesses?

Particular strengths of the CBGS cohort design include the rich collection of biological data and samples, including on maternal metabolism during pregnancy, environmental and dietary exposures and infant and breast milk samples. Information was obtained on both parents, and involvement of fathers meant that it was possible to analyse DNA trios, from mother, father and infant.

Multiple collaborations have enabled diverse laboratory analyses, including state-of-the-art mass spectrometry for novel metabolite and lipid profiling in infancy. As reflected by a dearth of reported data on hormone and metabolite levels between birth and mid-childhood, a major strength of CBGS is the collection of biological samples (largely by DBS sampling) from 3 months of age, as well as the large number of breast milk samples and DNA trios.

Limitations of CBGS include its single-site design and prolonged recruitment over an 8-year period. From birth to age 2 years, there was a 34% attrition rate which is comparable to other UK longitudinal cohorts. There are advantages and disadvantages of its South Cambridgeshire setting. The cohort is almost exclusively White Caucasian, which reduces confounding but also limits generalizability to other settings and populations. Additionally, the cohort has high rates of exclusive breastfeeding, compared with the whole of the UK. However, this improves the power for differences between breastfed and formula-fed infants to be observed.

A limitation of the body composition assessments is the absence of lean mass estimation, other than by extrapolation from skinfold thicknesses. With increasing evidence supporting the importance of the first few months of life as a critical period for long-term outcomes, rather than just the first year of life, ^22–24^ it would have been beneficial to include more frequent assessments, particularly in very early infancy.

## Can I get hold of the data? Where can I find out more?

Anonymized data are available to other investigators through collaborative agreements, and the CBGS co-investigators welcome formal or informal proposals and will consider these at their bimonthly meetings. Please contact Dr Carlo Acerini [cla22@cam.ac.uk].

## CBGS in a nutshell

- The CBGS is a prospective, observational pregnancy and birth cohort, primarily investigating antenatal and postnatal determinants of infancy growth and development.
- A total of 2229 pregnant women were recruited at approximately 12 weeks of gestation from one tertiary hospital in Cambridge, UK; 1658 newborns entered the infancy follow-up, born between 2001 and 2009.
- Detailed information collected during pregnancy included a first-trimester blood sample, oral glucose tolerance test and questionnaire data for demographics, medical history and environmental and dietary exposures.
- Infants were seen newborn and at 3, 12, 18 and 24 months of age, with longitudinal anthropometric measures, dried blood spot samples and questionnaire data. Breast milk and genetic data are also available.
- Any researcher interested in collaborating with the CBGS should contact Dr Carlo Acerini [cla22@cam.ac.uk].

## Funding

The Cambridge Baby Growth Study has been supported by the European Union Framework V, the World Cancer Research Foundation International, the Medical Research Council, the NIHR Cambridge Comprehensive Biomedical Research Centre, the Newlife Foundation for disabled children, the Mothercare Group Foundation, Mead Johnson Nutrition, the Evelyn Trust, the Wellbeing of Women, Diabetes UK and a collaborative research grant from the European Society for Paediatric Endocrinology.
